# The Lone Inventor: Low Success Rates and Common Errors Associated with Pro-Se Patent Applications

**DOI:** 10.1371/journal.pone.0033141

**Published:** 2012-03-21

**Authors:** Kate S. Gaudry

**Affiliations:** Harvard Law School, Cambridge, Massachusetts, United States of America; Kaohsiung Chang Gung Memorial Hospital, Taiwan

## Abstract

A pro-se patent applicant is an inventor who chooses to represent himself while pursuing (“prosecuting”) a patent application. To the author's knowledge, this paper is the first empirical study addressing how applications filed by pro-se inventors fare compared to applications in which inventors were represented by patent attorneys or agents. The prosecution history of 500 patent applications filed at the United States Patent and Trademark Office were analyzed: inventors were represented by a patent professional for 250 of the applications (“represented applications”) but not in the other 250 (“pro-se applications”). 76% of the pro-se applications became abandoned (not issuing as a patent), as compared to 35% of the represented applications. Further, among applications that issued as patents, pro-se patents' claims appear to be narrower and therefore of less value than claims in the represented patent set. Case-specific data suggests that a substantial portion of pro-se applicants unintentionally abandon their applications, terminate the examination process relatively early, and/or fail to take advantage of interview opportunities that may resolve issues stalling allowance of the application.

## Introduction

Patents offer a reward for what has been done (discovering an invention) and an incentive for what is left to do (developing and marketing the invention). Specifically, a patent owner can prevent others from making, using, or selling the claimed invention. If the inventor chooses to develop and market the invention himself, the patent can thus be used to ward off competition. If the inventor believes that a company is better situated to develop and market the idea, he may present his invention to the company and propose a licensing arrangement. His patent can protect him against the possibility that the company will forego compensating him while pursuing the idea nonetheless. Thus, patents offer a unique protection to individuals, in that they allow individuals to pursue collaborative efforts to develop their idea.

However, focusing on a patent's rewards alone provides an incomplete picture of benefits that the patent system provides to individuals. Two other questions must be considered: (1) what are the costs associated with pursuing a patent; and (2) what is the probability that such a pursuit will lead to issuance of a patent? If patent-pursuit costs are very high, or if patent issuance rates is very low, then it would appear as though the net value provided by the patent system to individual inventors is low.

To receive a patent, an applicant must file an application with the United States Patent and Trademark Office (PTO) and convince an examiner that the application meets statutory patent requirements. The application includes a specification that must describe the invention in sufficient detail to enable another person of skill in the art to make and use the invention [1]. The application must also include one or more claims that identify the subject matter regarded as the invention [2]. After the application is filed, it is assigned to an examiner who assesses whether the application meets the statutory patent requirements [3]. Frequently, the examiner issues an Office Action setting forth one or more rejections identifying why the application allegedly does not meet the statutory patent requirements. The applicant may respond in an Amendment or Response (“Amendment”) that explains why the rejections are inapposite and/or that amends the claims in an attempt to overcome the rejection. This exchange of communications is referred to as “prosecuting” a patent, in that an applicant is attempting to pursue an allowance of his application. The prosecution continues with exchanges of Office Actions and Amendments until the application is either allowed or abandoned.

An inventor may represent himself before the PTO. However, the PTO “strongly suggest[s]” using a registered patent attorney or agent [4]. Specifically, the Manual of Patent Examining Procedure states, “While an inventor may prosecute the application, lack of skill in this field usually acts as a liability in affording the maximum protection for the invention disclosed. Applicant is advised to secure the services of a registered patent attorney or agent to prosecute the application, since the value of a patent is largely dependent upon skilled preparation and prosecution.” [5]

Employing a professional for representation before the PTO carries a substantial cost: for example, the average attorney fees for merely preparing and filing an application is approximately $8,500–$15,500 [6]. The average cost for preparing and filing an Amendment is estimated to be between $2,200 and $4,500.

I compared the success and strategies of a sampling of applications filed by inventors alone (“pro-se applications”) to a sampling of applications for which inventors were represented by a patent attorney or agent (“represented applications”). Pro-se applications were more than twice as likely to be abandoned as compared to represented applications. Detailed analysis of the examination of pro-se applications identifies mistakes relatively common among pro-se applications that a pro-se inventor should be vigilant to avoid.

## Methods

I sent a request via the Freedom of Information Act to the PTO. I requested that the PTO identify, to the best of its ability, applications filed by pro-se inventors and available on the online Public Patent Application Information Retrieval (PAIR). A list of applications was provided for which no attorney docket number was identified in the application data sheet (ADS) accompanying the filing of the application [7]. I randomly sorted these applications to create “List 1”. Beginning at the top of List 1, I viewed the file history of the first application in PAIR. Each file history on PAIR includes electronic copies of all documents exchanged between the applicant and the PTO. I examined the ADS to confirm that no attorney or agent represented the inventor. I also scanned the file history to ensure that no power of attorney was filed before issuance of the first Office Action. If I concluded (1) that the inventor was represented, (2) that the patent application was not a utility patent application (and was instead a plant or design patent application), or (3) that the application was still being examined, I continued to the next listed application. Otherwise, I collected prosecution data for the application as described below. I continued down the sorted list in this manner until I identified 250 pro-se applications. The application numbers of these pro-se applications are listed in Table S1.

Next, I created “List 2” by adding one to each application number in List 1. Beginning at the top of this list, I viewed the file history of the first listed application in PAIR. If the ADS or a power of attorney indicated that a patent professional represented the inventor, the application was a utility application, and examination of the application was complete, I collected prosecution data for the application as described below. Otherwise, I continued on to the next application number in List 2. In this manner, 250 represented applications were identified. The application numbers of these represented applications are listed in Table S1.

For each identified pro-se and represented application, the following data was identified using the file history available using PAIR:

Whether a Notice to File Missing Parts, a Notice of Incomplete Application, a Notice of Omitted Item(s), or a Notice to File Corrected Application Papers was issued (issuance of any of these four Notices were equated to a Formality-Difficulty Indicator: Application Filing for calculations presented in Table 1; further related discussion is presented in the Results section.);
The types of rejections present in the first issued Office Action (a rejection under 35 U.S.C. 112(2) was equated to a Formality-Difficulty Indicator: Claims for calculations presented in Table 1; further related discussion is presented in the Results section);
Whether a Request for Continued Examination (RCE) and/or a Notice of Appeal was filed;
The total number of issued Office Actions (excluding Advisory Actions and Ex parte Quayle Actions; this number includes, e.g., an Office Actions filed after a Request for Continued Examinations);
Whether a notice of returned mail was recorded;
Whether a notice of a Non-Compliant Amendment was issued (issuance of this notice was equated to a Formality-Difficulty Indicator: Response to Office Action for calculations presented in Table 1; further related discussion is presented in the Results section);
Whether a Petition to Revive was filed;
Whether one or more interviews were conducted, and, if so, who initiated the interview (if an Interview Summary was present in the file history or if an interview was mentioned in an Examiner's Notice of Allowance, then it was determined that an interview was conducted. I determined whether the Examiner or Applicant initiated the interview based on the context of the interview summary or description);
The final outcome: allowance or abandonment; and
Whether personalized advice was offered in the first Office Action (if the first Office Action included advice about new potentially allowable claims or specific amendment strategies, the application was identified as having personalized advice. Examiner's recommendations to obtain representation or Examiner's indications of allowable claims were separately noted).

**Table 1:** Examination events and final results of pro-se and represented applications.

	Pro-Se	Represented
**Abandonment Rate**	76.4%^**^	34.8%^**^
**Unintentional Abandonment Indicators**		
*Probability of Mail Returned to PTO*	0.8%^**^	8.1%^**^
*Probability of Filing a Petition to Revive*	1.2%	5.3%
**Formality-Difficulty Indicators**		
*Application Filing*	51.0%^**^	23.6%^**^
*Claims*	64.9%^**^	26.9%^**^
*Response to Office Action*	42.4%^**^	6.4%^**^
**Probability of Responding to Indicated Office Action**		
*First Action*	41.2%^**^	85.6%^**^
*Second Action*	32.2%^**^	78.7%^**^
**Claim-Specific Advice or Allowable/Allowed Claims in First Office Action**	10.6%	19.0%
**RCE and/or Appeal Filed**	3.2%^**^	19.6%^**^

Table cells show percentages of pro-se applications (middle column) or represented applications (right columns) for which the left-column-indicated event occurred. The remaining cells show the average number of claims or words-per-independent-claim in analyzed pro-se patents or represented patents. For each of these variables, a two-by-two contingency table was analyzed using Fisher's exact test using GraphPad Software to identify a preliminary p-value. Per the conservative Bonferroni correction, each preliminary p-value was then divided by ten, as ten comparisons were performed. Two stars after the presented percentages indicate that the corrected p-value was significant to a level of p<0.01.

Throughout the manuscript, I frequently report a percentage of the pro-se applications and a percentage of the represented applications that met a particular criterion (e.g., had a final “abandonment” outcome). To assess whether the data sets were significantly different in any such regard, a two-by-two contingency table was analyzed to obtain a preliminary p-value using Fisher's exact test using GraphPad Software. As shown in Table 1, this type of analysis was performed ten times (with respect to ten different criterion). To conservatively estimate significance, each preliminary p-value was divided by ten per the Bonferroni correction.

Within each data set, 57 patented applications were identified. In the pro-se data set, this included all applications that issued as a patent. In the represented data set, I sequentially went through the list of applications (which, recall, was in a randomized order) in the data set until I identified 57 applications that issued as a patent. For each patent issuing from one of these patented applications, I identified:

The total number of claims;
The number of independent claims; and
The average number of words per independent claim.

For each variable, data from the two data sets were compared using a two-sample t-test. In this analysis, p-values were not adjusted.

## Results

The results below first characterize the final outcome of the pro-se and represented applications. To offer explanations for the marked differences in patent-prosecution success, I then present analysis of the prosecution strategies of the applications. Tables 1–2 summarize many of the results.

**Table 2:** Statistics of claims issued in patented pro-se and represented applications.

	Pro-Se	Represented
**Average Number of Total Claims**	10.9^*^	14.2^*^
**Average Number of Independent Claims**	1.8	2.1
**Average Mean Independent-Claim Word Count**	263^*^	181.7^*^

All of the patented pro-se applications were analyzed (N = 57) and a same number of patented represented applications were analyzed. Within each data set, the number of claims, the number of independent claims, and the average independent-claim word count were identified. Table cells show the within-data-set average of these variables. A two-sample t-test was performed for each variable to determine whether there was a significant difference between the data sets. One star after the presented percentages indicates that the un-adjusted p-value was significant to a level of p<0.05.

### Three-Quarters of Pro-Se Applications, but only One-Third of Represented Applications, are Abandoned

The abandonment rate of pro-se applications (76.4%) was significantly higher than the abandonment rate of represented applications (34.8%). Thus, more than twice as many pro-se applications were abandoned as compared to represented applications (76.4% versus 34.8%).

Potentially, the discrepancy in the allowance rates may be attributable to pursuit of patents protecting different types of inventions. All of the analyzed applications were utility applications (rather than design or plant applications), but nevertheless, the subject matter of the applications may be a stronger predictor of allowance rate than pro-se status. To address this possibility, I noted each application's assignment to one of eight technology centers (TCs). The TCs are differentiated by numbers and include the following eight centers: ***TC-1600***: Biotechnology and Organic Chemistry; ***TC-1700***: Chemical and Materials Engineering; ***2100***: Computer Architecture, Software, and Information Security; ***TC-2400***: Computer Networks, Multiplex Communication, Video Distribution, and Security; ***TC-2600***: Communications; ***TC-2800***: Semiconductors, Electrical and Optical Systems and Components; ***TC-3600***: Transportation, Construction, Electronic Commerce, Agriculture, National Security and License & Review; and ***TC-3700***: Mechanical Engineering, Manufacturing, Products [8]. The PTO assigns the application to a single TC based on a general classification of application's claimed subject matter.

A binary logistic regression analysis was performed to test whether pro-se applications were significantly less likely to be allowed as compared to represented applications despite any differences in TC assignments. The MATLAB GLM fitting function glmfit() was used, setting the distribution to “binomial” and the linkage to “logit” (The Mathworks, Natick, MA). The model is defined as:
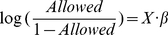
(1)
*Allowed* is an outcome variable and is defined as “1” when the application was allowed and “0” when it was abandoned. *β* is a vector of coefficients, each coefficient corresponding to one of the columns in *X*.

*X* is a matrix of explanatory variables. The first column of *X* is set to “1”, corresponding to a constant across applications. The second column of *X* is a *Pro_Se* variable, which was defined as “1” when the application was characterized as a pro-se application and “0” when the application was characterized as a represented application.

In a first analysis, *X* includes only the above-described six columns. In a second analysis, *X* also includes columns related to TC assignments. In this analysis, the third through sixth columns of *X* relate only to TC assignments – the third column (*TC_1_*) is set to “1” if the application is assigned to TC-1600 or TC-1700 and to “0” otherwise. The fourth column (*TC_2_*) is similarly defined but for assignments to TC-2100 or TC-2400, the fifth column (*TC_3_*) for assignments to TC-2600 or TC-2800, and the sixth (*TC_4_*) for assignments to TC-3600 or TC-3700. Each application is assigned to one technology center, and thus, only one of columns 3–6 is set to “1” for each application. In the second analysis, *X* also includes four interaction columns, which identify when an application was *both* a pro-se application and assigned to one of the TC categories defined above. In this analysis, elements of column seven are interaction elements and are the products of elements in column 1 and column 3. Similarly, elements in columns eight through ten are the products of elements in column 1 and one of columns 4–6.

Table 3 shows results of the logistic-regression analysis. The middle column shows estimates of coefficients obtained when only the *Pro_Se* variable was considered, without regard to any *TC* variable. As shown, whether the application was a pro-se application or represented application was a very significant predictor (p<0.01) as to whether the application would be allowed. The negative sign of the *Pro_Se* coefficient indicates that pro-se applications were less likely to be allowed than represented applications.

**Table 3:** Logistic Regression Results.

	Allowance – TC not considered	Allowance – TC considered
***Constant***	0.63^**^	0.85^**^
	(0.13)	(0.18)
***Pro_Se***	−1.80^**^	−2.00^**^
	(0.20)	(0.24)
***TC_1_***		−0.89^*^
		(0.42)
***TC_2_***		0.18
		(0.52)
***TC_3_***		−0.61
		(0.58)
***TC_4_***		−0.11
		(0.41)
***Pro_Se • TC_1_***		0.064
		(0.93)
***Pro_Se • TC_2_***		0.60
		(1.04)
***Pro_Se • TC_3_***		0.030
		(1.13)
***Pro_Se • TC_4_***		0.21
		(0.83)

A logistic regression analysis was performed to assess whether the predictive variables shown in the left column significantly contributed to allowance-rate predictions. Two regressions were performed: technology-center variables and interaction variables were not considered in the first (results shown in the middle column) but were in the second (results shown in the right column). The cell values identify regression coefficients, and standard errors are shown in parentheses. Variables significantly contributing to the allowance-rate prediction are marked with one or two stars following the regression coefficient: one star indicates that *p*<0.05 and two stars indicate that *p*<0.01.

The right column shows estimates of coefficients obtained when *TC* variables were also considered. The *TC_1_* variable (i.e., whether the application was assigned to TC-1600: “Biotechnology and Organic Chemistry” or TC-1700: “Chemical and Materials Engineering” versus another TC) significantly contributed to the allowance prediction at the p<0.05 level. None of the other *TC* variable nor any of the interaction variables (between the *Pro_Se* variable and one of the *TC* variables) significantly contributed to the allowance prediction. Notably, the analysis considered only a general subject-matter categorization of applications, and more detailed subject-matter inquiry could yield different results. Nevertheless, the analysis suggests that allowance-rate discrepancies between the data sets are largely attributable to whether an applicant represented himself or hired a professional, and not due to differences in fields of invention (e.g., semiconductor applications versus mechanical-engineering application). Thus, other statistics reported herein only relate to a comparison between pro-se-application data and represented-application data are reported, without further distinguishing TC assignments.

### Pro-Se Patents Appear to be Narrower than Represented Patents

Mere issuance of a patent does not generally indicate that an applicant “successfully” prosecuted the patent application. Rather, the value of any issued patent must also be considered. Each patent includes one or more claims that define the scope of the protection afforded by the patent. If the scope is exceedingly narrow, then competitors could easily work around the inventor's invention and sell similar products. Thus, the patent would be of little value. If the claims were very broad, then the inventor could potentially monopolize a market or demand royalty payments. In this case, the patent would be very valuable.

Defining claim scope is a difficult task, as it requires knowledge of the relevant industry. One study analyzed which patent characteristics were most prevalent among patents that were maintained for a long period of time (and therefore presumably more valuable). Two variables that were correlated with long patent maintenance were claims' word counts and the number of claims per patent [9]. Simplistically, this result makes sense. Shorter claims would seemingly include fewer limitations and would cover more devices or methods. Similarly, a claim set including a large number of claims would seem to protect more embodiments, as each claim within the set must be of a unique scope. Therefore, for each set of analyzed patents, I identified the average number of words in the independent claims and the number of claims as proxies for the value of the patent.

**Figure 1:**
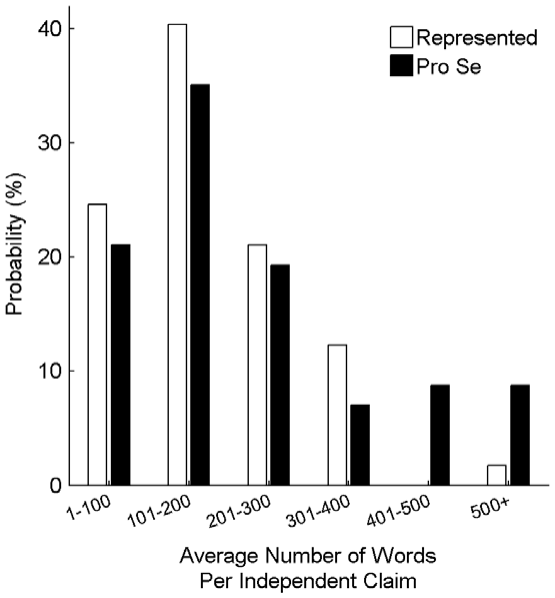
Distributions of independent-claim word counts. The graph shows the distribution of the average word count across independent claims in represented applications (white bars) and in pro-se applications (black bars).

Figure 1 shows the distribution of the average independent-claim word counts for represented and pro-se patents. Across the represented patents, the mean independent-claim-averaged word count was 182 words. The mean independent-claim-averaged word count for pro-se applications was significantly higher (p<0.05), at 263 words.

**Figure 2:**
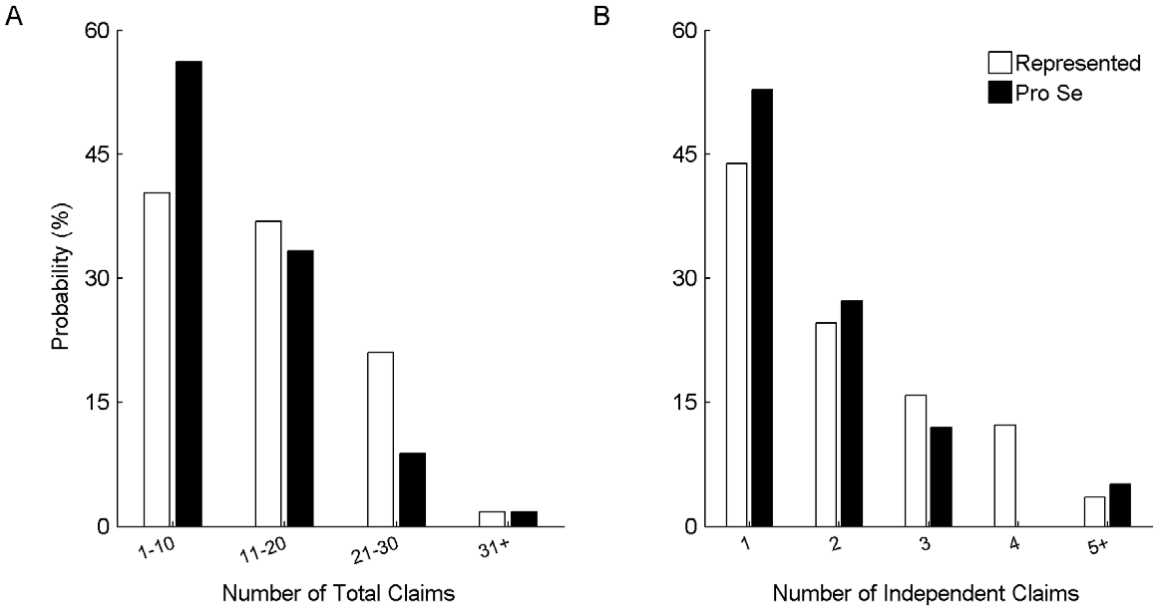
Distributions of number of total and independent claims. ***A:*** The distribution of the number of total claims across represented patents (white bars) and across pro-se patents (black bars). ***B:*** The same as in (A) but for the number of independent claims.

Figure 2A shows the distribution of the number of claims across the represented and pro-se patent data sets. On average, pro-se patents include significantly fewer claims than do represented patents (p<0.05; 11 claims for pro-se patents versus 14 claims for represented patents). Within a claim set, an independent claim does not depend on any other claims. Meanwhile, a dependent claim depends on an independent claim and adds another limitation [10]. Thus, independent claims are broader than dependent claims. Figure 2B therefore also shows the distribution of the number of independent claims for the patent data sets. On average, pro-se patents include 1.8 independent claims, while represented patents include 2.1 independent claims. (This difference was not significant; p = 0.15.)

### Pro-Se Inventors Appear to be Relatively Unfamiliar with PTO Requirements

For each application, I analyzed the communications exchanged between the PTO and the applicant in an effort to understand why pro-se applicants were fairing worse than represented applicants. Not only does this analysis identify several common mistakes, but it also provides a general picture of how well a pro-se applicant can navigate through the patent system.

#### Non-compliance with PTO Formalities

Statutes and PTO rules set forth a number of requirements that must be met before an application may be examined by the PTO [11]. When an application is initially filed, it must include a number of components: the full application, an oath or declaration in which the inventor states that he believes the named inventor/s were the first inventor/s of the claimed subject matter, fees, and a translation, if necessary [12]. If one or more of these are not submitted or not complete at the time of filing, the PTO will issue a Notice to File Missing Parts, a Notice of Incomplete Application or a Notice of Omitted Item(s) [13], [14]. Further, if application documents fail to conform to technical requirements (e.g., if the application's abstract is not on a separate page or claims are not consecutively numbered), a Notice to File Corrected Application Papers is issued [15]. The Applicant may then submit the missing item or a corrected item, though the filing date may then be the date that the missing item was received [16].

Thus, issuance of any of the above-identified notices indicates that the applicant failed to meet one or more filing requirements set forth in the patent rules or statutes. In some instances, such a filing deficiency may have been intentional. For example, a company may recognize that a patent application must be filed before a disclosure. The application may be filed before a declaration is ready to be filed, such that the application can be filed as quickly as possible. However, in other instances, these notices indicate that the filing entity was unfamiliar with the filing requirements.

For each application in the pro-se data set and for each application in the represented data set, I determined whether one or more of these notices were issued. Such a notice was issued in 51% of the pro-se applications and in 24% of the represented applications (a significant difference: p<0.01).

If the PTO accepts an application, the Examiner then reviews the claim/s to ensure that they meet the statutory requirements. One statutory requirement is the “112(2)” requirement, stating that the claims must “particularly point … out and distinctly claim … the subject matter which the applicant regards as his invention.” [17] The Examiner may, for example, assert that claims do not meet this requirement if there is a lack of antecedent basis of a limitation (e.g., if the claim refers to “the lever” without first introducing “a lever”); or a claim includes the phrase “for example” or “such as” [18]. In some instances, even a skilled professional may draft claims not meeting the 112(2) requirement (e.g., due to hurried drafting). In other instances, 112(2) rejections are indicative of a lack of understanding of how claims must be worded.

I identified the percentage of applications within each data having a first Office Action issued with one or more 112(2) rejections. 65% of the pro-se applications included a 112(2) rejection, compared to 27% of the represented applications (a significant difference: p<0.01).

Statutes and rules also identify requirements for filing an Amendment [19]. For example, if the Amendment includes an amendment to any single claim, all of the claims must be presented, and the status of each claim must be identified using one of seven identifiers [20]. If the Amendment does not conform to the requirements, a Notice of Non-Compliant Amendment is issued and the applicant must correct the Amendment before it will be forwarded to an examiner. A Notice of Non-Compliant Amendment was issued in 42% of the pro-se applications and only 6% of the represented applications (a significant difference: p<0.01).

**Figure 3:**
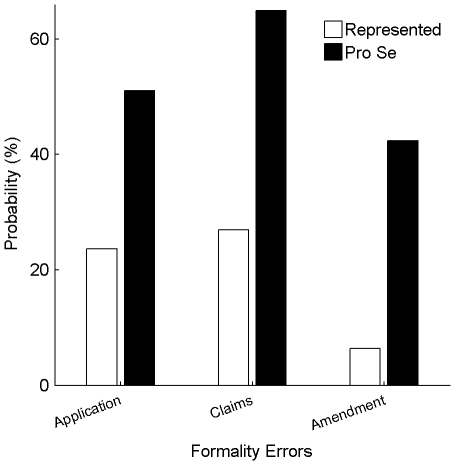
Prevalence of failure to conform to formal requirements. The graph shows the probability among represented applications (white bars) and among pro-se applications (black bars) that the PTO issued (1) a Notice to File Missing Parts, a Notice of Incomplete Application, a Notice of Omitted Item(s), or a Notice to File Corrected Application Papers (first set of bars); (2) a first Office Action with a 112(2) rejection (second set of bars); or (3) a Notice of a Non-Compliant Amendment (third set of bars).

Figure 3 summarizes the results of the above formality-related queries. Pro-se applications were more than twice as likely to receive each of the above-described formality-related notices and rejections. Upon receiving one of these notices or rejections, an applicant has the opportunity to correct the identified error. However, if he fails to do so within the specified time period, the application is abandoned. Further, regardless of whether these errors actually led to abandonment, this data is informative as to pro-se applicants' general understanding of patent-prosecution requirements.

#### Unintentional Abandonment

One potential explanation for the relatively high pro-se-application abandonment rate is that pro-se inventors unintentionally abandon their application. After an application is filed, the applicant is responsible for responding to PTO communications (e.g., by supplementing incomplete applications, responding to rejections in Office Actions, or paying issuance fees). If the applicant fails to respond to a PTO communication within the prescribed time, the application is abandoned.

It would be exceedingly difficult to identify the precise fraction of abandoned applications that were unintentionally abandoned. However, it is informative to identify the fraction of applications in which a Petition to Revive was filed, which may be filed when an application was unavoidably or unintentionally abandoned [21]. This petition was filed in 5.3% of pro-se applications and 1.2% of the represented applications. It is likely that these percentages under-estimate pro-se applicants' unintentional abandonments, as the applicants may have been unaware of the opportunity to revive the application or may have been deterred by the petition fee ($810 for small inventors [22]).

Another data point suggestive of the occurrence of at least some unintentional abandonments is the probability of mail being returned to the PTO. Returned mail was recorded in approximately one-twelfth (8.4%) of the pro-se applications. This is an order of magnitude higher than the represented-applications' mail-return frequency (0.8%). The difference in mail-return occurrence was significant (p<0.01). It is the responsibility of the applicant to notify the PTO of an address changes [23]. Therefore, failure to update one's address with the PTO may lead to abandonment even if an applicant is thereafter unaware of communications sent by the PTO to him. These variables suggest that a substantial percentage of pro-se applicants unintentionally abandoned their applications.

### Few Pro-Se Applicants Engage in Lengthy Patent Prosecution

**Figure 4:**
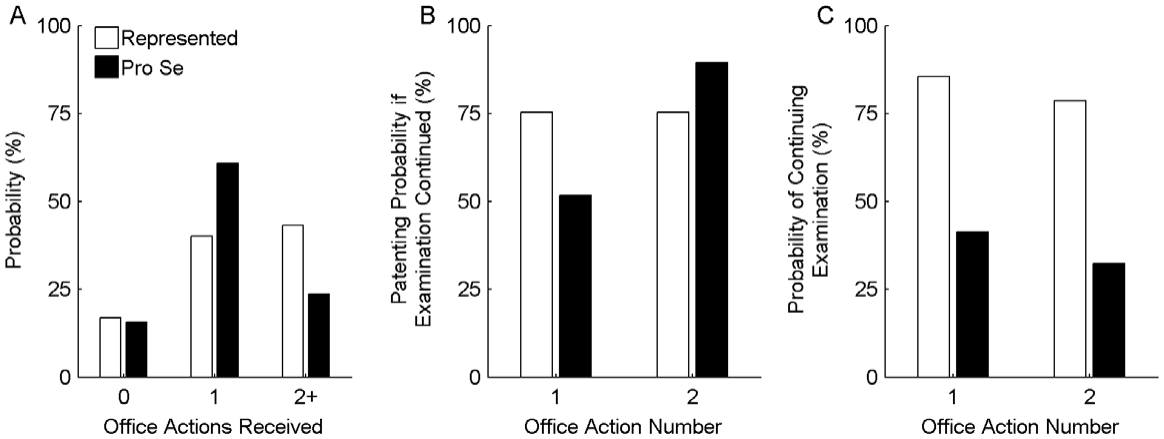
Probability and success of responding to Office Actions. ***A:*** The distribution of the total number of Office Actions received across represented applications (white bars) and across pro-se applications (black bars). ***B:*** The probability that an application was ultimately allowed if an applicant responded to a first (left bars) or second (right bars) received Office Action in a manner that continued examination (e.g., by filing a compliant response and/or by filing a Request for Continued Examination if necessary). As shown in (A), not all applications received a first and/or second Office Action, so the data shown is from the subset of applications for which such an Action was received. ***C:*** The probability that an applicant responded to a first (left bars) or second (right bars) received Office Action in a manner that continued examination.

As described above, examination of an application includes a series of communications between the examiner and applicant. Specifically, the examiner typically rejects an application in an Office Action, the Applicant files an Amendment to respond to the rejections, the examiner may issue another Office Action, etc. Figure 4A shows the distribution of the number of Office Actions received per application. Among pro-se applications, only 24% of the applications received two or more Office Actions.

Upon receiving an Office Action, an applicant determines whether examination of the application will continue. Filing an Amendment (potentially accompanied by a Request for Continued Examination, which is discussed in more detail below) would accomplish this objective. Figure 4B shows the probability of an application being ultimately allowed when the applicant responded in such a manner to a particular Office Action. As shown, when a pro-se applicant received and responded to a first Action, there was a 52% of ultimate allowance. Nevertheless, pro-se applicants were significantly less likely to respond to each Office Action as compared to represented applicants (p<0.01 for probabilities of responding to first Office Actions and p<0.01 for probabilities of responding to second Office Actions). (Figure 4C.) For example, only 41% of pro-se applicants (versus 86% of represented applicants) chose to respond to a first Office Action. Within this group, only 32% of pro-se applicants receiving another Office Action replied to it (versus 79% of represented applicants).

An applicant is not able to indefinitely pursue patent prosecution based only on his initial filing fee. The initial fee provides only for prosecution slightly beyond issuance of a “final” Office Action. An examiner is to characterize a second or subsequent Office Action as “final” if it does not include any new ground of rejections that is necessitated neither by a claim amendment nor by a new piece of art identified by the applicant as being relevant [24]. Thus, any second Office Action is frequently characterized as final. Upon receiving a final Office Action, the applicant may attempt to persuade the examiner that the rejections are without merit [25]. At this point, the applicant does not have a right to amend any of the finally rejected claims, so the persuasion must be based purely on arguments [26]. If the arguments are unsuccessful or the applicant chooses to forego this opportunity, he may: file a Request for Continued Examination (RCE) to continue examination of the application and introduce any new claim amendments, if desired [27], or he may appeal the rejection to the Board of Patent Appeals and Interferences by filing a Notice of Appeal [28], each of which is accompanied by an additional fee. If the rejection is not withdrawn and no RCE or Notice of Appeal is filed, the application becomes abandoned.

**Figure 5:**
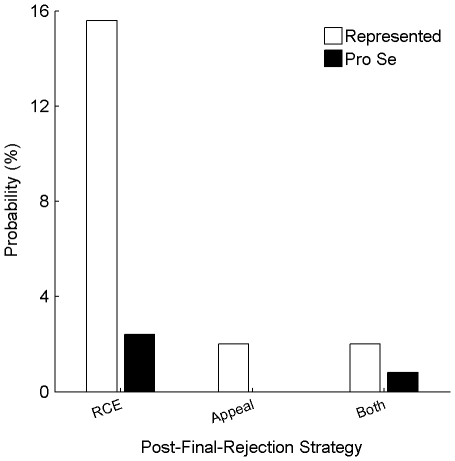
Prevalence of post-final rejection strategies. The graph shows the probability among represented applications (white bars) and among pro-se applications (black bars) that an applicant filed a Request for Continued Examination (RCE), a Notice of Appeal, or both.

Figure 5 shows the prevalence of these post-final-rejection strategies. In total, an RCE and/or a Notice of Appeal were filed in 20% of the represented applications. Meanwhile, only 3% of the pro-se applications employed one of these strategies (a significant difference: p<0.01).

Therefore, it appears as though the fate of pro-se applications depended largely on examiners' initial analysis of the application. If the examiner rejected the application, pro-se applicants frequently immediately abandoned the application. The expense or lack of awareness of the post-final-rejection strategies may explain why these strategies were rarely used in pro-se applications. However, pro-se applicants frequently did not even respond to an initial Office Action, which is very rarely final. Therefore, pro-se applicants frequently forewent free opportunities to attempt to overcome rejections based on arguments or amendments.

### Pro-Se Applicants Rarely Receive or Solicit Advice from Examiners

When it appears as though an application is a pro-se application, the PTO's rules indicate that “the examiner may suggest to the applicant that it may be desirable to employ a registered patent attorney or agent” if there is appears to be patentable subject matter [29]. Additionally, if the examiner believes that the inventor is representing himself and that there is patentable subject matter in the application, the examiner is to include one or more draft claims in the Action and indicate that such claims would be allowable if the inventor amended the claim set to incorporate them [30]. For all patent applications (i.e., represented and pro-se), an examiner is to identify any allowed claims and any claims that would be allowed if the claims' were amended to correct its form [31]. For example, if the examiner concluded that independent claim 1 was not obvious over cited art, but dependent claim 2 was not, he may identify claim 2 as being allowable if rewritten to be in independent form.

**Figure 6:**
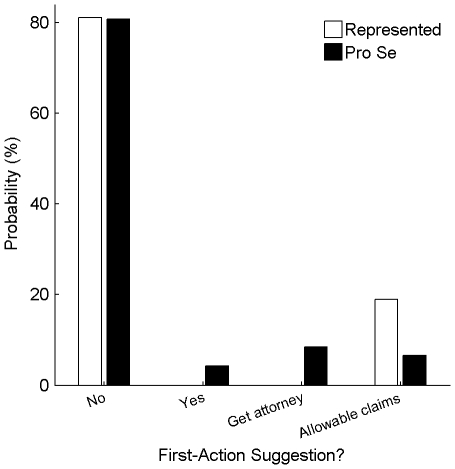
Prevalence of examiner advice in first Office Actions. The graph shows the probability among represented applications (white bars) and among pro-se applications (black bars) that the a first Office Action included (1) no advice at all (first group of bars); (2) content-specific advice, such as suggested new claims or claim amendments (second group of bars); (3) advice for an inventor to seek the assistance of a patent attorney or agent (third group of bars); or (4) identification of allowable or allowed claims (fourth group of bars).

Figure 6 shows how frequently various forms of advice were provided by the examiner in first Office Actions. In approximately 80% of the applications, the examiner offered no advice in the first Office Action. Allowable or allowed claims were identified in 19% of first Office Actions for the represented applications and 7% of first Office Actions for the pro-se applications. Among the pro-se applications, 8% of the first Office Actions included advice to hire a patent attorney or agent, and only 4% of the applications included specific advice in terms of amendments that may lead to allowance of the claims. The probability of receiving a first Office Action with allowable/allowed claims or claim-specific advice was 19% for represented applications an approximately half that (11%) for pro-se applications, though this difference was not significant (p>0.05).

Another opportunity to seek an examiner's advice and opinions is through an “interview”, during which an applicant, attorney or agent can discuss matters related to an application (e.g., its patentability) with an examiner [32]. An interview may be conducted in-person, over the phone, or through email. Either an applicant (or his representative) or an examiner may initiate the interview.

The Director of the PTO has characterized interviews as being effective at quickly resolving issues [33]. However, it is unclear whether pro-se inventors are even aware of the opportunity of interviewing. While Office Actions include the examiner's contact information, the applicant may not understand that this information is any more than a communication formality. I identified the percentage of applications within each data set for which one or more interviews were conducted during examination. Additionally, based on summaries and dates of the interviews presented in official communications from the PTO and Amendments, I attempted to determine whether the examiner or the applicant initiated the interview. In some instances, more than one interview was conducted during examination, with both the examiner and the applicant initiating one or more of the interviews.

**Figure 7:**
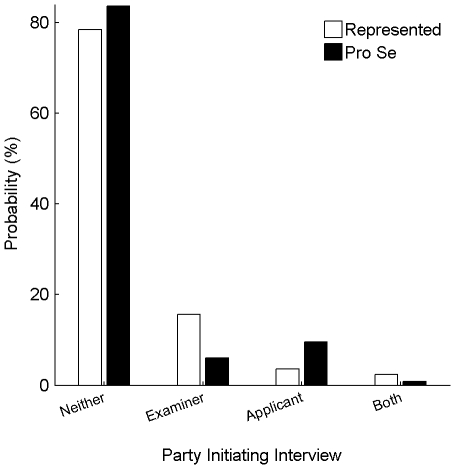
Prevalence of interviews. The graph shows the probability among represented applications (white bars) and among pro-se applications (black bars) that one or more interviews was conducted during examination of each application. Among applications in which one or more interviews were conducted, the data is further divided based on whether the examiner, the applicant (or the applicant's representative), or both the examiner and applicant initiated one or more interview.

Figure 7 shows the interview probabilities. The vast majority of the cases were not interviewed: 84% of pro-se applications and 78% of represented applications. Examiners were more likely to initiate interviews for represented applications, which they did for 18% of the applications. These interviews were frequently to request permission from the applicant for the examiner to enter an Examiner's Amendment of the claims, such that he could issue a Notice of Allowance allowing the amended claims. Among the pro-se applications, applicants were the primary interview initiator. In total, only 16% of the pro-se applications were interviewed.

## Discussion

As described above, a patent can provide protection to individuals seeking to fully develop and market their invention or to license the idea to a company better equipped for the entailed manufacturing and marketing. However, this protection depends critically on two factors: (1) whether a patent application actually issues into an enforceable patent; and, (2) if so, whether the claims cover the invention and foreseeable design-arounds (similar products or methods sufficiently different from the claimed subject matter to escape liability). The above-presented analysis suggests that pro-se patent applicants are less likely to achieve desirable patenting results. (Figures 1 and 2.) While pro-se inventors saved money initially, the 76% abandonment rate indicates that most wasted the filing fees paid to the PTO.

### Potential Causes of Pro-Se Applications' High Abandonment Rate

#### Failure to Understand PTO Requirements

A variety of the above-presented data suggests that pro-se applicants generally lack an understanding of patent-related requirements and opportunities, which may at least partly explain the abandonment discrepancy between pro-se and represented applicants. The text accompanying Figure 3 identifies a number of formal requirements for an initial patent-application filing or for responses to Office Actions. Formality-related notices and claim-indefiniteness rejections are issued relatively frequently among the pro-se applications, suggesting that many applicants do not know of or understand these requirements. (See Figure 3 and associated text.) Further, mail was returned to the PTO in 8% of the pro-se applications, suggesting that some applicants were unaware of their responsibility to update their address with the PTO.

Petitions to Revive were filed in 5% of the pro-se applications. While some of these applications were revived, others were not. I predict that this percentage greatly under-estimates the incidence of unintentional abandonments, as pro-se applicants may have been unaware of the petitioning opportunity or unwilling to pay the accompanying fee.

#### Early Surrender

Another potential cause of the high abandonment rate is that the applicants unjustifiably gave up hope of securing a patent [34]. Only 41% and 32% of pro-se applicants responded to a first or second Office Action in a manner that continued examination. These percentages are less than half of those identified among represented applicants. (Figure 4.) Applicants may be unaware that “rejections” in Office Action do not indicate the application's final state: the rejections can be traversed and/or claims can be amended. Indeed, Figure 4 shows that traversals of Office Actions frequently lead to allowances even among the pro-se applications. However, to successfully traverse a rejection, an applicant must realize that he can respond to an Office Action with arguments or amendments, and he must be able to recognize a flaw in the stated rejections or to identify an amendment likely to overcome the rejections. The low response rate may be at least partly because an applicant may assign merit to even a faulty rejection that includes boilerplate case law, PTO regulations and a myriad of cited references.

Identifying an appropriate strategy following receipt of a “final” Action may be particularly confusing to a pro-se applicant. The designation of the Action of “final” may suggest to the Applicant that it is not possible to further pursue the application. Only 3% of the pro-se applications included a post-final-rejection strategy of an RCE or Notice of Appeal. (Figure 5.) I hypothesize that many pro-se applicants are unaware of these strategies.

Thus, a pro-se applicant may abandon an application too easily based on confusion of the merits of the rejections and unawareness of available response options.

#### Desired Claims Cannot Meet Patent Requirements

A third potential cause of the high abandonment rate is that no legal argument or action (e.g., claim amendment) would be sufficient to put a pro-se application in condition for allowance. While claims can be amended during patent prosecution, the amendments are constrained by the information originally disclosed in the specification [1]. Thus, original or amended claims not enabled or fully described in the original specification cannot be allowed [1]. A poorly drafted application may prevent even a skilled patent professional from convincing an examiner to allow any claims. Additionally, the invention sought to be protected by the claims must be novel [35], non-obvious [36], and of patentable subject matter [37]. An applicant attempting to patent an age-old product will not succeed in his efforts.

#### Cost

A fourth potential cause of the high abandonment rate is that pro-se applicants may be unable to justify further monetary or time expenses involved in pursuing a patent. After an application is filed, an applicant can respond to Actions for no additional fee. After issuance of a final Action, the applicant has only the following choices: (1) persuade the examiner that the rejections are in error; (2) persuade the examiner to enter claim amendments overcoming the rejections; (3) file an RCE to continue examination of the application; or (4) file a Notice of Appeal [38]. If the applicant does not accomplish one of the above tasks, the application becomes abandoned [38]. For a small entity, the cost of filing an RCE or a Notice of Appeal is $405 or $270, respectively [22]. The applicant may decide that continued prosecution is not worth this additional expense.

However, these costs are only applicable when a final Action has been received. Meanwhile, most Actions are abandoned before receipt of a final Action. (See Figure 4.) Even in these circumstances, an applicant may choose to abandon an application because he cannot justify foreseeable additional time involved in continuing to pursue the patent. If a pro-se applicant is unfamiliar with claim rejections, responding to an Action may appear to require researching the types of rejections, analyzing cited art, and drafting an appropriate Amendment. This task could easily involve a substantial time investment for someone new to the patent system.

### Considerations for Inventors

As stated above, the PTO currently recommends that pro-se inventors seek the advice of a registered patent attorney or agent [4], [29]. While patent attorneys and agents typically charge substantial amounts to draft and prosecute patent applications, inventors must consider the value of their services. Admittedly, pro-se applicants may initially save money by foregoing representation. However, the abandonment rate for pro-se applications is over twice that for represented applications, suggesting that pro-se applicants are more than twice as likely to have completely wasted their filing-fee and time investments. Additionally, even if a pro-se applicant succeeds in securing a patent, pro-se patents include fewer and longer claims as compared to represented patents (Figures 1 and 2), suggesting a narrower scope of protection. Thus, pro-se applicants should at least consider that pro-se applications typically fare worse than represented applications when deciding whether to hire a patent attorney or agent.

If an applicant decides to prosecute an application himself, he should thoroughly familiarize himself with the patent requirements. Initially, he must ensure that the application is drafted well. A poorly drafted application may prevent the applicant from supporting any valuable claims [1]. One useful reference is the Inventors Resources page on the PTO website, which sets forth information about patent requirements and search strategies [39].

Further, the pro-se applicant should be aware that critical requirements accompany more than just the application. He should ensure that each filing made during the prosecution (e.g., filing the initial application or filing a response to an Office Action) complies with all requirements. The applicant may wish to consult the Manual of Patent Examining Procedure, which is provided on the PTO's website and sets forth the requirements for various filings [40]. The applicant should be aware that responses to PTO notices or Office Actions must be made within the identified time period. Failing to respond within this time period causes the application to become abandoned.

Additionally, an inventor has several personal resources at his disposal. He may call the PTO's Inventor's Assistance Center at 800-785-9199 to clarify any confusion about what must be filed [41]. Once the application has been filed and assigned to an examiner, he may also contact the examiner with questions. This may be most valuable when attempting to determine how to respond to a rejection of the application. If the applicant identifies himself as a pro-se inventor, the examiner will likely help explain the rejections set forth in the Action and the options available to the applicant in responding to the rejections.

Finally, pro-se applicants should recognize that receiving rejections in Office Actions is extremely common. Of course, an applicant will likely be unable to overcome rejections if the initial application is very poorly drafted. However, if the applicant has a good-faith belief that the rejections are erroneous or could be overcome by amending the claims in a manner supported by the original specification, he should attempt to respond to the rejection accordingly. Again, calling the examiner to discuss the situation may provide insight into appropriate options.

### The Larger Picture of Pro-Se Representation

This article presents an analysis of pro-se inventors' efforts to self-navigate through the patent system. Currently, pro-se inventors fare worse than represented inventors. However, this issue extends beyond the boundaries of patent law. In patent law, an inventor has the option as to whether to pursue a patent or not and may (following a pursuit decision) subsequently decide upon a self- or professional-representation strategy. In other legal contexts (e.g., family law, criminal law, etc.), involvement with the law may not be optional. Factors such as costs or dissatisfaction with counsel may persuade a litigant to represent himself. Thus, it may be even more critical to assess whether the legal system is accessible to an unrepresented person.

A number of studies have quantitatively assessed the fate of pro-se litigants in other settings. For example, one 2006 study analyzed state-court and federal-court data to compare outcomes received by pro-se criminal defendants as compared to represented criminal defendants [42]. It was found that a small percentage of defendants represent themselves (primarily due to dissatisfaction with counsel) but, interestingly, they do not suffer adverse outcomes. While it is relieving to see that a criminal's defendants' fate is not dependent upon a representation status, these types of cases do not appear to represent the majority of self-representation occurrences in the legal arena.

For example, surveyed Arkansas circuit-court judges indicated that self-representation was most frequently observed for Divorce and Domestic Abuse cases [43]. They further reported that they perceived self-representation to negatively affect the litigants and the trial's efficiency. The judges also expressed concern about favoritism: at least some pro-se litigants relied on the judge for advice about what to do during proceedings. Judges were then faced with balancing the need to provide the pro-se litigant with necessary information without advocating for the litigant. Many judges supported the availability of approved forms, explanatory brochures and toll-free help line, and a website to assist the pro-se applicants and improve efficiency. Interestingly, similar resources are already available to pro-se inventors, though these inventors may be unaware of such resources.

Thus, it appears as though a number of the results that are presented herein are common to many legal fields. Specifically, self-representation may negatively affect legal outcomes. Self-representing people may be unfamiliar with formal legal requirements. Thus, initial documents filed (e.g., at the PTO or with a court) may not meet the requirements and may delay proceedings. A self-representing person may access an insufficient number of resources in order to provide necessary assistance about requirements and strategies to progress through the legal proceedings. Examiners and judges may struggle to both inform a self-representing person about legal requirements and options and refrain from advocating for the person.

### Considerations for PTO

As described above, the PTO already has many resources available to assist a pro-se inventor. The USPTO website includes many pages describing the patent process and requirements, and inventors may call an Inventors' Assistance Center with questions. Nevertheless, it appears as though many pro-se inventors do not have a thorough understanding of the patent process. It may be productive if the PTO would provide a pro-se inventor with at least some of this information in a personal communication.

For example, the PTO may adjust its filing forms (e.g., its Application Data Sheet) to require an inventor to indicate whether he is representing himself, or the PTO may estimate this characteristic based on other available data (e.g., a correspondence address or a lack of an attorney-docket number). Information about online resources, the toll-free Inventors' Assistance phone number, patent requirements, and the examination process may then be immediately sent to suspected or known pro-se inventors following filing. For example, it appears as though it may be very useful for a pro-se applicant to understand that a rejection in a first or even a “final” Office Action is not indicative of the application's final state. It also appears as though at least some pro-se applicants may be unaware of prosecution strategies such as RCEs and appeals. Further, the PTO could stress the importance of updating mailing addresses to avoid any inadvertent abandonments due to returned mail. Alternatively or in addition, similar information may be provided in the first Office Action.

Should an Examiner suspect or know that an applicant is representing himself, an examiner-initiated interview may be of great assistance to the applicant in clarifying any misunderstandings about prosecution options. It is recognized that it is not an Examiner's job is not equivalent to an attorney's job, and that the Examiner must continue to impartially enforce the law. Thus, it is my opinion that Examiners should use communications to attempt to impress realistic understandings of the law and operation of the patent system upon a pro-se inventor.

For example, I believe that a pro-se applicant should be made aware that issuances of an Office Actions with claim rejections is common, and that applications are frequently ultimately allowed even if it is initially rejected (e.g., after filing one or more Amendments responsive to one or more Office Actions). However, the applicant should also be aware that amendment and response strategies are limited based on the originally filed application and that a substantial portion of applications never issue into patents. The applicant may then be better informed in deciding whether and how to respond to a received Office Action.

### The New PTO Effort: The Pro Bono Pilot Program

The PTO has recently launched a new pilot program that offers great promise to pro-se inventor: a Pro Bono Pilot Program [44]. The program launched on June 8, 2011. The USPTO partnered with LegalCORPS of Minneapolis (a non-profit organization). Currently, the program offers assistance to Minnesota-resident inventors who have filed a patent application that has been rejected by the PTO. The PTO notes that the program may soon expand to assist other inventors and small businesses that meet a financial need level.

It's the author's view that expansion of this program would greatly assist pro-se inventors and make the patent system more accessible to the individual inventors. This type of program has the potential to offer an individual inventor, not only assistance during the prosecution stages, but also prior to filing an application. Prosecution strategies and claiming options are tied to a specification as originally filed. Thus, drafting a strong initial application drastically improves later prosecution strategies. If these types of programs become widespread and well known, a pro-se applicant may be able to better understand legal and practical elements tied to patent requirements and patent prosecution.

### Conclusion

In conclusion, inventors representing themselves typically fare worse than inventors represented by patent professionals: their applications are less likely to be allowed to issue, and any issuing patent is likely of relatively narrow scope. The data suggests that one cause of this discrepancy may be pro-se applicants' unfamiliarity with the patent requirements. Therefore, an inventor wishing to represent himself should carefully research patent and filing requirements, be aware of prosecution responsibilities and filing deadlines, and use available resources (e.g., examiner interviews). Existing PTO-provided resources and new pilot programs may improve the probability that a pro-se inventor may successfully understand and access the patent system.

## Supporting Information

Table S1 **Application numbers of each application included in the pro-se or represented data sets.** The middle and right columns show the application numbers of each application analyzed for the pro-se-application data set and represented data set, respectively.
(DOCX)
