# Supplementary material for: The Lone Inventor: Low Success Rates and Common Errors Associated with Pro-Se Patent Applications
Source: PLoS One. 2012 Mar 21;7(3):e33141. doi: 10.1371/journal.pone.0033141 (PMC3310007; doi:10.1371/journal.pone.0033141)
Supplement: Table S1 — Application numbers of each application included in the pro-se or represented data sets. The middle and right columns show the application numbers of each application analyzed for the pro-se-application data set and represented data set, respectively. (DOCX) [file pone.0033141.s001.docx]

***Table S1***

|  | **Pro-Se Applications** | **Represented Applications** |
| --- | --- | --- |
| 1 | 11319155 | 11725808 |
| 2 | 11651759 | 11369786 |
| 3 | 11740929 | 11725348 |
| 4 | 11642812 | 11319156 |
| 5 | 10906771 | 11158122 |
| 6 | 12077616 | 11070997 |
| 7 | 11087417 | 11273347 |
| 8 | 11300361 | 11740930 |
| 9 | 11105853 | 11718632 |
| 10 | 10905509 | 11642813 |
| 11 | 11850673 | 11899071 |
| 12 | 11237704 | 11087418 |
| 13 | 11684661 | 11300362 |
| 14 | 11467653 | 11642280 |
| 15 | 11208181 | 11269347 |
| 16 | 11233784 | 11520961 |
| 17 | 11618027 | 11105854 |
| 18 | 11567771 | 12080374 |
| 19 | 11881387 | 10905510 |
| 20 | 12075930 | 11055236 |
| 21 | 11145088 | 11052727 |
| 22 | 11397383 | 11182508 |
| 23 | 11710633 | 11237705 |
| 24 | 11160555 | 11684662 |
| 25 | 11588156 | 11478156 |
| 26 | 12284513 | 11064556 |
| 27 | 11160040 | 11133735 |
| 28 | 11826051 | 11618028 |
| 29 | 11331563 | 11567772 |
| 30 | 12217207 | 11576113 |
| 31 | 11285267 | 11545052 |
| 32 | 11377782 | 11579456 |
| 33 | 11784144 | 12075931 |
| 34 | 11803291 | 11450445 |
| 35 | 11973996 | 11639089 |
| 36 | 11052621 | 11106491 |
| 37 | 11160069 | 11160556 |
| 38 | 11705117 | 11588157 |
| 39 | 11177767 | 11655237 |
| 40 | 11890457 | 11464262 |
| 41 | 11161182 | 12455726 |
| 42 | 12072668 | 11880921 |
| 43 | 12383408 | 11160041 |
| 44 | 11818939 | 11826052 |
| 45 | 11101676 | 11483889 |
| 46 | 11949042 | 11331564 |
| 47 | 11751038 | 11267689 |
| 48 | 11365312 | 12217208 |
| 49 | 11345767 | 11277778 |
| 50 | 11639998 | 11285268 |
| 51 | 11527687 | 11377783 |
| 52 | 12074309 | 11985295 |
| 53 | 11546759 | 11542361 |
| 54 | 11805382 | 11784145 |
| 55 | 11711069 | 11206718 |
| 56 | 11346438 | 11052622 |
| 57 | 11247726 | 11160070 |
| 58 | 11897423 | 11705118 |
| 59 | 12002511 | 11177768 |
| 60 | 11405311 | 11165301 |
| 61 | 11090994 | 11161183 |
| 62 | 11669170 | 11351452 |
| 63 | 11117001 | 11751039 |
| 64 | 11247385 | 11146324 |
| 65 | 11446987 | 11365313 |
| 66 | 11274646 | 11453670 |
| 67 | 11292765 | 11832689 |
| 68 | 12215393 | 11050880 |
| 69 | 11654132 | 11345768 |
| 70 | 11788790 | 11511197 |
| 71 | 11409672 | 11639999 |
| 72 | 11235483 | 12271898 |
| 73 | 11430628 | 11163706 |
| 74 | 11806083 | 11244007 |
| 75 | 11183938 | 11546760 |
| 76 | 11347239 | 11805383 |
| 77 | 11941760 | 11440806 |
| 78 | 11980055 | 11639818 |
| 79 | 12387776 | 11247727 |
| 80 | 11430143 | 11150380 |
| 81 | 11160815 | 11588944 |
| 82 | 11171989 | 12092270 |
| 83 | 11308527 | 11405312 |
| 84 | 11799600 | 11982860 |
| 85 | 11161519 | 11818970 |
| 86 | 11287431 | 11073369 |
| 87 | 11710700 | 11669171 |
| 88 | 11434011 | 11356481 |
| 89 | 11328935 | 12070083 |
| 90 | 11288463 | 11247386 |
| 91 | 11833259 | 11274647 |
| 92 | 11218161 | 11408285 |
| 93 | 11429771 | 12124691 |
| 94 | 11170000 | 11124732 |
| 95 | 11394576 | 11292766 |
| 96 | 11709965 | 11323732 |
| 97 | 12048222 | 11650108 |
| 98 | 12136762 | 11584964 |
| 99 | 11705590 | 12229629 |
| 100 | 11037947 | 11187680 |
| 101 | 11221336 | 11654133 |
| 102 | 11702232 | 10905478 |
| 103 | 11341378 | 11409673 |
| 104 | 11648068 | 11971105 |
| 105 | 10908300 | 11483250 |
| 106 | 11497773 | 11235484 |
| 107 | 12380921 | 11806084 |
| 108 | 11497440 | 11449907 |
| 109 | 11306332 | 11184365 |
| 110 | 11164812 | 12006472 |
| 111 | 11724752 | 11725839 |
| 112 | 11047530 | 11319341 |
| 113 | 12114796 | 11821970 |
| 114 | 12384284 | 11980056 |
| 115 | 10535251 | 11451670 |
| 116 | 11440772 | 11053536 |
| 117 | 11502718 | 11369346 |
| 118 | 11900584 | 11115234 |
| 119 | 11647833 | 11612547 |
| 120 | 11161454 | 11160816 |
| 121 | 11149375 | 11308528 |
| 122 | 11162937 | 11161520 |
| 123 | 11162620 | 11232929 |
| 124 | 10907967 | 11283208 |
| 125 | 11674019 | 11287432 |
| 126 | 11445692 | 12056648 |
| 127 | 11967260 | 11710701 |
| 128 | 12075292 | 11311101 |
| 129 | 11117750 | 11328936 |
| 130 | 11311708 | 11065139 |
| 131 | 11825402 | 11288464 |
| 132 | 11247719 | 11188594 |
| 133 | 11490106 | 11303123 |
| 134 | 11078818 | 11094511 |
| 135 | 11979422 | 11218162 |
| 136 | 12636719 | 11343817 |
| 137 | 11704120 | 11429772 |
| 138 | 11474869 | 11504120 |
| 139 | 11119812 | 11170001 |
| 140 | 11099309 | 11208501 |
| 141 | 11244506 | 11064452 |
| 142 | 11973616 | 11808587 |
| 143 | 11669819 | 11416611 |
| 144 | 11543656 | 11261984 |
| 145 | 11542018 | 11529643 |
| 146 | 11804390 | 12048223 |
| 147 | 10549845 | 11472634 |
| 148 | 10580634 | 11741059 |
| 149 | 11655597 | 11872397 |
| 150 | 11197689 | 11055856 |
| 151 | 11455528 | 11637646 |
| 152 | 12080590 | 12346445 |
| 153 | 11657611 | 11130900 |
| 154 | 11649059 | 11221337 |
| 155 | 12152164 | 12321792 |
| 156 | 11512130 | 12321397 |
| 157 | 11088624 | 11487879 |
| 158 | 11894276 | 11157410 |
| 159 | 12075626 | 11376853 |
| 160 | 11447542 | 11094144 |
| 161 | 11516861 | 11293784 |
| 162 | 11506939 | 10908301 |
| 163 | 11588154 | 11481164 |
| 164 | 12216650 | 11590034 |
| 165 | 11500214 | 12150700 |
| 166 | 11713938 | 11630531 |
| 167 | 12148498 | 11679168 |
| 168 | 11809445 | 12009782 |
| 169 | 11503767 | 11358229 |
| 170 | 12069587 | 11057437 |
| 171 | 11646629 | 11469940 |
| 172 | 11379212 | 11164813 |
| 173 | 12150979 | 11161458 |
| 174 | 11106083 | 11724753 |
| 175 | 12164036 | 11899186 |
| 176 | 12077675 | 11527318 |
| 177 | 11036270 | 12102744 |
| 178 | 11204485 | 10535252 |
| 179 | 11046909 | 11487566 |
| 180 | 11299419 | 11440773 |
| 181 | 11239046 | 11977639 |
| 182 | 11789599 | 11900585 |
| 183 | 11307125 | 12009477 |
| 184 | 11985747 | 11811746 |
| 185 | 11318233 | 11198720 |
| 186 | 11529149 | 11162938 |
| 187 | 11505653 | 11162621 |
| 188 | 10906297 | 10907968 |
| 189 | 11633104 | 11674020 |
| 190 | 11702749 | 11445693 |
| 191 | 11355157 | 11967261 |
| 192 | 11408742 | 11878180 |
| 193 | 11084585 | 12011661 |
| 194 | 11265487 | 11104856 |
| 195 | 11974214 | 11974760 |
| 196 | 12007274 | 12017668 |
| 197 | 11542326 | 11117751 |
| 198 | 11032862 | 11825403 |
| 199 | 11083238 | 11606535 |
| 200 | 11394800 | 11247720 |
| 201 | 12032685 | 11169037 |
| 202 | 11530464 | 12096580 |
| 203 | 11646928 | 12002515 |
| 204 | 12157437 | 11490107 |
| 205 | 12029145 | 11078819 |
| 206 | 11516407 | 11979423 |
| 207 | 12216649 | 11248850 |
| 208 | 11467173 | 11899746 |
| 209 | 11176750 | 11167697 |
| 210 | 11491368 | 11404315 |
| 211 | 11729445 | 11887076 |
| 212 | 11708870 | 11317264 |
| 213 | 12660651 | 11704121 |
| 214 | 11896143 | 11119813 |
| 215 | 10596807 | 12291955 |
| 216 | 11900247 | 11332760 |
| 217 | 11603588 | 11311236 |
| 218 | 11725390 | 11244507 |
| 219 | 11224356 | 12218431 |
| 220 | 11430142 | 12244573 |
| 221 | 11303815 | 11383143 |
| 222 | 11158829 | 11669260 |
| 223 | 11941079 | 11700684 |
| 224 | 12329538 | 11669820 |
| 225 | 11419504 | 11543657 |
| 226 | 11031495 | 11450971 |
| 227 | 11399699 | 11804391 |
| 228 | 11986751 | 11999292 |
| 229 | 11490276 | 11049900 |
| 230 | 11323170 | 11273555 |
| 231 | 11975876 | 11455529 |
| 232 | 11699821 | 12168213 |
| 233 | 12214778 | 11322196 |
| 234 | 11056148 | 11346685 |
| 235 | 11507249 | 11657612 |
| 236 | 11034627 | 12325951 |
| 237 | 11626376 | 12576266 |
| 238 | 11515269 | 11473629 |
| 239 | 12458427 | 11649060 |
| 240 | 11443802 | 11088625 |
| 241 | 11698235 | 11402711 |
| 242 | 11301814 | 12075627 |
| 243 | 11234854 | 11447543 |
| 244 | 11044767 | 11161445 |
| 245 | 11642080 | 11506940 |
| 246 | 11053464 | 11901923 |
| 247 | 11491888 | 11062726 |
| 248 | 11163974 | 12172413 |
| 249 | 12151951 | 10906952 |
| 250 | 11816894 | 11235890 |

**Table S1: Application numbers of each application included in the pro-se or represented data sets.** The middle and right columns show the application numbers of each application analyzed for the pro-se-application data set and represented data set, respectively.
